# Residual effect of narasin on feed intake and rumen fermentation characteristics in Nellore steers fed forage-based diet

**DOI:** 10.1093/tas/txad027

**Published:** 2023-03-10

**Authors:** Letícia Carolina Bortolanza Soares, Rodrigo S Marques, Alexandre Vaz Pires, Vinicius Alves Cruz, Makayla Anne Ogg, Arnaldo Cintra Limede, Paulo César Gonzales Dias Junior, Isabela Jorge dos Santos, Rhaíssa Garcia de Assis, Vinícius N Gouvêa, Evandro Maia Ferreira, Daniel Montanher Polizel

**Affiliations:** Department of Nutrition and Animal Production, FMVZ, University of São Paulo, Pirassununga, SP 13635-000, Brazil; Department of Animal and Range Science, Montana State University, Bozeman, MT 59717, USA; Department of Nutrition and Animal Production, FMVZ, University of São Paulo, Pirassununga, SP 13635-000, Brazil; Department of Animal Science, College of Agriculture “Luiz de Queiroz”, University of São Paulo, Piracicaba, SP 13418-900, Brazil; Department of Animal and Range Science, Montana State University, Bozeman, MT 59717, USA; Department of Animal and Range Science, Montana State University, Bozeman, MT 59717, USA; Department of Nutrition and Animal Production, FMVZ, University of São Paulo, Pirassununga, SP 13635-000, Brazil; Department of Animal Science, College of Agriculture “Luiz de Queiroz”, University of São Paulo, Piracicaba, SP 13418-900, Brazil; Department of Animal Science, College of Agriculture “Luiz de Queiroz”, University of São Paulo, Piracicaba, SP 13418-900, Brazil; Department of Animal Science, College of Agriculture “Luiz de Queiroz”, University of São Paulo, Piracicaba, SP 13418-900, Brazil; Texas A&M AgriLife Research and Extension Center, Amarillo, TX 79106, USA; Department of Animal Science, College of Agriculture “Luiz de Queiroz”, University of São Paulo, Piracicaba, SP 13418-900, Brazil; Department of Nutrition and Animal Production, FMVZ, University of São Paulo, Pirassununga, SP 13635-000, Brazil; Department of Animal Science, College of Agriculture “Luiz de Queiroz”, University of São Paulo, Piracicaba, SP 13418-900, Brazil

**Keywords:** additive, ionophores, ruminal parameters, washout

## Abstract

This study aimed to evaluate the residual effect of narasin on intake and ruminal fermentation parameters in Nellore cattle fed a forage-based diet. Thirty rumen-cannulated Nellore steers [initial body weight (**BW**) = 281 ± 21 kg] were allocated to individual pens in a randomized complete block design, with 10 blocks and 3 treatments, defined according to the fasting BW at the beginning of the experiment. The animals were fed a forage-based diet containing 99% Tifton-85 haylage and 1% concentrate. Within blocks, animals were randomly assigned to one of three treatments: (1) forage-based diet without addition of narasin (CON; *n* = 10), (2) CON diet plus 13 mg of narasin/kg DM (**N13**; *n* = 10), or (3) CON diet plus 20 mg of narasin/kg DM (**N20**; *n* =10). The experiment lasted 156 d and was divided into two periods. The first period lasted 140 d and consisted of the daily supply of narasin. In the second period (last 16 d), the animals were not supplemented with narasin when the residual effect of the additive was evaluated. The treatments were evaluated by linear and quadratic orthogonal contrasts. The results were reported as least square means and the effect was considered significant when *P* ≤ 0.05. No treatment × day interaction was identified for dry matter intake (*P* = 0.27). There was a treatment × day (*P* ≤ 0.03) interaction after narasin removal for the molar proportion of acetate, propionate, ac:prop ratio, and ammonia nitrogen. The inclusion of narasin decreased linearly (*P* < 0.01) the molar proportion of acetate (*P* < 0.01), and this effect persisted until day 5 after narasin withdrawal (*P* < 0.01). Narasin inclusion linearly increased the molar proportion of propionate (*P* < 0.04), and linearly decreased (*P* < 0.01) ac:prop ratio up to 5 d after removing narasin from the diets. No treatment effects were observed (*P* > 0.45) on days 8 and 16 after the withdrawal. Narasin linearly decreased ammonia nitrogen up to 1 day after withdrawal (*P* < 0.01). In conclusion, the use of narasin for a prolonged period (140 d) resulted in a residual effect on rumen fermentation parameters after the removal of the additive from the diets.

## INTRODUCTION

Grazing beef cattle production systems frequently face several obstacles to meet the nutrient requirements of the animal, including seasonal variation in forage availability, nutritive value, and sward structure, affecting daily nutrient intake and performance (de Souza et al., 2017; Kunkle et al., 2000). In addition, several studies have reported the ineffectiveness of supplementing grazing animals and difficulties in predicting individual intake of the supplement and additives by animals (Bagley et al., 1988; Bowman and Sowell, 1997). However, despite all the challenges of a grazing production system, there are several possibilities to optimize animal performance, including pasture management (Johnstone-Wallace and Kennedy, 1944), the use of supplements (Kunkle et al., 2000), and the manipulation of rumen fermentation through the use of additives (Marques and Cooke, 2021; Russell and Strobel, 1989).

Ionophores are the main additives used in ruminant nutrition and can be included in multiple supplements to improve feed efficiency. These feed additives optimize rumen fermentation routes by increasing propionate and reducing ruminal proteolysis and methane production (Bergen and Bates, 1984; Duffield et al., 2012). Several ionophores are available on the market, whereas animal performance response may vary depending on dosage (Bretschneider et al., 2008), type of ionophore (Limede et al., 2021), diet (Bretschneider et al., 2008), forage (Lana and Russell, 1997), and frequency of supply (Soares et al., 2021).

Narasin is an ionophore used for grazing animals in South America, and recent research shows that it improves performance of beef cattle fed forage-based diets without affecting dry matter intake (**DMI**) of protein or mineral supplements (Limede et al., 2021; Polizel et al., 2020). These results highlight the potential of using this additive in grazing systems as a nutritional alternative in low-consumption mineral and protein supplements that benefit grazing cattle performance (Limede et al., 2021; Polizel et al., 2017).

Although the mechanisms of action and effects of narasin are well established in the literature, little is known about how ruminal metabolism works with less frequent consumption of the additive and its impact on cattle during withdrawal. Marques and Cooke (2021) reported that the results of continuous use of ionophores and its effects after withdrawal help beef cattle producers define supplementation strategies to optimize rumen fermentation parameters in grazing systems. Nevertheless, research is warranted to validate the persistence efficacy of each ionophore over a long period of use on ruminal parameters. The information on the withdrawal effect of narasin allows for optimizing the practical use of this additive. It is also of great importance for experiments in reversal (e.g., Latin square, crossover design). Moreover, it would help determine a minimum withdrawal interval between supplementation periods. Therefore, we hypothesize that after narasin withdrawal, there is a residual effect of narasin on intake and ruminal fermentation parameters in Nellore beef cattle fed a forage-based diet. Thus, this study aimed to evaluate the residual effect of narasin on intake and ruminal fermentation parameters in Nellore beef cattle fed a forage-based diet.

## MATERIALS AND METHODS

The study was carried out at the University of São Paulo (USP/ESALQ), Piracicaba, São Paulo, Brazil (22° 42ʹ 24″ S and 47° 37ʹ 53″ W). All animals used were treated according to the experimental protocols reviewed and approved by the Institutional Committee for the Use and Care of Animals at ESALQ/USP (University of São Paulo, protocol #2093090119). Narasin was supplemented for 140 d and was described by Polizel et al. (2020).

### Animals, Experimental Design, and Diets

At the beginning of the first experimental period (Polizel et al., 2020), thirty-two rumen-cannulated *B. indicus* Nellore steers [initial body weight (**BW**) = 281 ± 21 kg] were assigned to individual pens (tie-stall concrete-surface system; 2.5 × 4.5 m, with a feed bunk and waterer) in a randomized complete block design according to their initial shrunk BW (after 16 h of water and feed withdrawal). Within each block (*n* = 10), steers were randomly assigned to 1 of the 3 treatments: (1) forage-based diet without the addition of narasin (**CON**; *n* = 10), (2) CON diet plus 13 mg of narasin/kg of DM (Zimprova; Elanco Animal Health, São Paulo, Brazil; N13; *n* = 10), 3) 20 mg narasin/kg DM (Elanco Animal Health; N20; *n* = 10).

Throughout the experimental period, the steers of all treatments received 99% Tifton-85 haylage (*Cynodon dactylon* spp.) and 1% concentrate composed of a 50:50 mixture of ground corn:soybean hulls (as-fed basis), which was used as a delivery vehicle for supplying narasin (N13 and N20). The animals from the CON treatment received the same proportion of concentrate, whereas without narasin. Haylage was chopped daily with a vertical mixer (Mixer VM8B, DeLaval International AB, Rumba, Sweden). The concentrate was supplied to each steer individually and daily before offering haylage to prevent the small amount of supplement from mixing with the forage and compromising the immediate ingestion of the concentrate. All steers had *ad libitum* access to haylage, water, and mineral mixture throughout the experimental period. The mineral mixture was offered in a separate feed bunk from the haylage and concentrate. The mineral supplement (Bellmais; Trouw Nutrition; Mirassol, SP, Brazil) used contained 178 g/kg Ca, 60 g/kg P, 17 g/kg S, 135 g/kg Na, 5,000 mg/kg Mg, 650 mg/ kg Cu, 500 mg/kg Mn, 2400 mg/kg Zn, 48 mg/kg I, 38 mg/kg Co, 12 mg/kg Se, and 1000 mg/kg Fe. Narasin was not included in the mineral mixture to ensure that the exact amount, based on the individual forage intake, would be supplied and consumed by the steers.

The first period lasted 140 d (Polizel et al., 2020) and consisted of the daily offer of concentrate without narasin for the CON treatment and concentrate with narasin in treatments N13 and N20. The second period lasted 16 d, and all animals received the control treatment (without including narasin). Ruminal fermentation parameters were evaluated regarding the withdrawal effect of narasin during this period.

### Sampling, Laboratory Analyses, and Measurements

Throughout the experiment, forage intake, concentrate, and total DMI were recorded daily by collecting and weighing refusals (only forage). Treatment amounts were calculated daily based on the previous day’s individual total forage DMI. Steers were fed treatments once daily (0800 hours) and had *ad libitum* access to haylage (0830 h), allowing up to 5% orts. Samples of haylage and supplements were collected daily, and analyzed for nutrient profile (Table 1).

**Table 1.** Nutritional profile of forage Tifton-85 (*Cynodon dactylon* spp.) and concentrate (corn and soybean hulls).

Item^1^	Haylage	Concentrate
Dry matter	42.4 ± 6.83	89.0 ± 1.25
Crude protein, % DM	8.56 ± 2.10	10.8 ± 0.80
Neutral detergent fiber, % DM	68.0 ± 4.66	39.7 ± 1.59
Hemicellulose, % DM	34.4 ± 3.37	13.2 ± 0.92
Acid detergent fiber, % DM	33.9 ± 2.53	26.6 ± 1.05
Ash, % DM	6.65 ± 0.89	3.39 ± 0.21
Ether extract, % DM	1.77 ± 0.20	3.13 ± 0.15

^1^Based on nutritional profile of each ingredient, which were analyzed via wet chemistry procedures (AOAC, 1990).

Ruminal fluid was collected in the second period of the experiment on days 0 (last day of narasin supply), 1, 3, 5, 8, and 16, after the removal of narasin (washout period), and 6 h after concentrate was offered. A representative rumen sample was collected via cannula from three points of the rumen (caudal, ventral, and cranial sac). The ruminal contents were filtered through layers of nylon cloth to obtain approximately 200 mL of ruminal fluid, and the solid phase was returned to the rumen. The pH of the rumen fluid was determined immediately after filtering with a pH meter (Digimed-M20; Digimed Analytical Instrumentation; Sao Paulo, SP, Brazil). Two 15 mL aliquots were obtained and stored for further analysis of ruminal ammonia concentration and short-chain fatty acid (SCFA) molar ratios (acetate, propionate, butyrate, isobutyrate, valerate, and isovalerate).

The forage samples and concentrate samples were dried in a forced ventilation oven at 60 °C for 72 h, ground in a Willey-type mill with a 1-mm sieve, and analyzed for DM at 105 °C (AOAC, 1990; method #934.01) and ash by incineration at 550 °C for 4 h (AOAC, 1990; method #942.05). Total N was determined using the Leco TruMac N according to AOAC (1990; method #968.06). The CP was obtained by multiplying the total N by 6.25. Sequential detergent fiber analyses were used to determine neutral detergent fiber (**NDF**; Van Soest et al., 1991) and acid detergent fiber (**ADF**; Goering and Van Soest, 1970) with an Ankom 2000 fiber analyzer (Ankom Tech. Corp., Macedon, NY, USA). Sodium sulfite and heat-stable α-amylase were included in the NDF analyses. Hemicellulose was calculated by the difference between NDF and ADF. The ether extract content was determined using an Ankom^XT15^ Extrator (Ankom Technology, Macedon, USA), according to AOAC (1990; method #920.29), using petroleum ether. For the determination of SCFA, 1.6 mL of ruminal fluid was centrifuged for 60 min at 15,000 × *g* at 4°C. The supernatant (0.4 mL) was transferred to a chromatography flask and 0.2 mL of 3:1 solution of metaphosphoric acid (250 mL/L) and formic acid (908 to 1,000 mL/L) were added. The internal standard was added to each vial (0.1 mL of 100 mM 2-ethyl-butyric acid). Analyzes were performed using the Agilent 7890A gas chromatograph equipped with flame ionization detection (7683B) and fused silica capillary column (J & W 19091F-112, Agilent Technologies, Santa Clara, CA, USA), 25 m long and 320 µm internal diameter. The total execution time of the chromatograph and the external calibration curve were described by Ferreira et al. (2016). Ruminal ammonia nitrogen concentration was determined by a colorimetric method (Chaney and Marbach, 1962) adapted for reading on a microplate (EON, BioTech Instruments, Winooski, VT, USA) with a 550 nm absorbance filter.

### Statistical Analysis

For all the analyzed variables, the animal was considered the experimental unit, and the data were submitted to the Levene test to verify the homogeneity of the variances, while the Shapiro–Wilk test was used to verify the normality of the residues and remove outliers. The data were analyzed using the PROC MIXED procedure of SAS (Version 9.4; SAS Inst. Inc.; Cary, NC). The Kenward–Roger approximation was used to determine the denominator df for the test of fixed effects. For all variables, the model statement contained the effects of treatment, block, day, and treatment × day interaction. Data were analyzed using animals(treatment) as the random variable. The covariance structure was first-order autoregressive, providing the best fit for these analyses according to the lowest Akaike Information Criterion Corrected (AICC). Treatments were evaluated by linear (L) and quadratic (Q) orthogonal contrasts. The effect of the day and treatment × day interaction was defined by the *F* test. Significance was set at *P* ≤ 0.05 and tendencies were determined if *P* > 0.05 and ≤ 0.10. Results are reported according to the main effects if no interactions were significant.

## RESULTS

**Figure 1.**
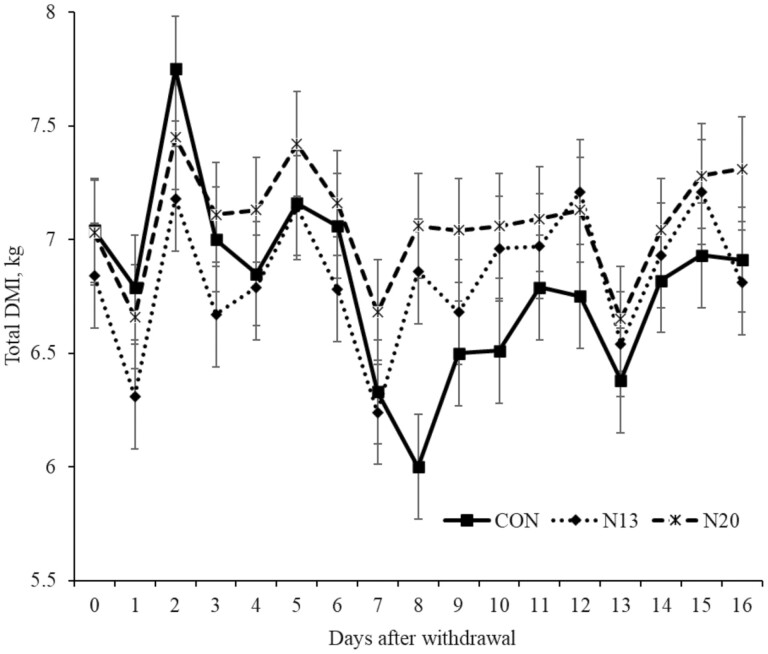
Total dry matter intake (DMI) during the narasin withdrawal period (16 d) of Nellore steers (*Bos indicus*) fed forage-based diet (*Cynodon dactylon* spp.) supplemented or not (**CON**; *n* = 10) with 13 mg of narasin/kg of DM (**N13**; *n* = 10), or 20 mg narasin/kg DM (**N20**; *n* = 10) for 140 d before narasin withdrawal. Day effect *P* < 0.01. Day 0 represents the last day of narasin supplementation described by Polizel et al. (2020).

Narasin was offered to the animals for 140 d (Polizel et al., 2020) before the withdrawal. After narasin withdrawal, no treatment effect was observed for forage intake (*P* > 0.17), concentrate intake (*P* > 0.08), and total DMI (*P* > 0.18; Figure 1). All analyzed variables had a day effect (*P* < 0.01) Table 2.

**Table 2.** Intake during narasin withdrawal (16 d) of Nellore steers (*Bos indicus*) fed forage-based diet (*Cynodon dactylon* spp.) supplemented or not (CON; *n* = 10) with 13 mg of narasin/kg of DM (N13; *n* = 10), or 20 mg narasin/kg DM (N20; *n* = 10) for 140 d (Polizel et al., 2020).

Item	Treatments^1^			SEM^2^	P-value^3^			
	CON	N13	N20		L	Q	Day	T × D
*After narasin withdrawal* ^4^								
Intake, kg/d								
Forage, kg	6.79	6.76	7.01	0.19	0.31	0.17	<0.01	0.39
Concentrate, g	63.47	63.42	64.47	1.38	0.08	0.16	<0.01	0.64
Total, kg	6.85	6.83	7.08	0.19	0.31	0.18	<0.01	0.39
Intake, %/BW	1.91	1.88	1.94	0.07	0.86	0.49	<0.01	0.70
Intake, g/kg BW^0.75^	83.26	81.98	84.33	2.48	0.83	0.44	<0.01	0.66

^1^The diets were supplied daily, and forage was offered *ad libitum* throughout the experimental period (days 0 to 156), CON: forage-based diet without addition of narasin, N13: CON diet plus 13 mg of narasin/kg of DM; N20: CON diet plus 20 mg narasin/kg DM as described by Polizel et al. (2020).

^2^SEM: standard error of the mean.

^3^Treatments were evaluated by linear (L) and quadratic (Q) orthogonal contrasts. Interaction between treatment and day (T × D).

^4^Period related to narasin withdrawal (16 d).

No treatment × day interactions were identified for the molar proportion of isobutyrate, butyrate, isovalerate, valerate, total SCFA, and pH (Table 3). There was a day effect for all analyzed variables (*P* < 0.01), except for isovalerate (*P* = 0.10) and ruminal pH (*P* = 0.60).

**Table 3.** Ruminal concentrations of short-chain fatty acids (SCFA) and pH during narasin withdrawal (16 d) of Nellore steers (*Bos indicus*) fed forage-based diet (*Cynodon dactylon* spp.) supplemented or not (**CON**; *n* = 10) with 13 mg of narasin/kg of DM (**N13**; *n* = 10), or 20 mg narasin/kg DM (**N20**; *n* = 10) for 140 d (Polizel et al., 2020).^1^

Item	Treatments^2^			SEM^3^	*P*-value^4^			
	CON	N13	N20		L	Q	Day	T × D^5^
SCFA, mM/100 mM								
Acetate	74.86	74.21	73.99	0.22	<0.01	0.68	<0.01	<0.01
Propionate	13.30	14.05	13.85	0.11	<0.01	0.01	<0.01	<0.01
Isobutyrate	0.84	0.85	0.87	0.02	0.21	0.48	<0.01	0.61
Butyrate	8.43	8.33	8.58	0.12	0.39	0.08	<0.01	0.06
Isovalerate	1.39	1.36	1.47	0.06	0.18	0.06	0.10	0.43
Valerate	1.19	1.18	1.20	0.03	0.99	0.66	<0.01	0.44
Total VFA, mM	97.79	105.90	102.87	3.02	0.10	0.13	<0.01	0.10
Ac:prop^6^	5.64	5.30	5.36	0.06	<0.01	0.04	<0.01	<0.01
pH	6.86	6.86	6.93	0.07	0.32	0.49	0.60	0.91
Ammonia, mg/dL	9.15	8.42	8.46	0.57	0.14	0.53	<0.01	0.03

^1^The second period (last 16 d of the experiment) was determined as the removal of narasin, where all steers were fed CON treatments (without narasin inclusion).

^2^The diets were supplied daily, and forage was offered *ad libitum* throughout the experimental period, CON: forage-based diet without addition of narasin, N13: CON diet plus 13 mg of narasin/kg of DM; N20: CON diet plus 20 mg narasin/kg DM as described by Polizel et al. (2020).

^3^SEM: standard error of the mean.

^4^Treatments were evaluated by linear (L) and quadratic (Q) orthogonal contrasts. The effect of the day and treatment × day interaction was defined by the *F* test.

^5^Interaction between treatment and day (T × D).

^6^Acetate:propionate ratio.

**Figure 2.**
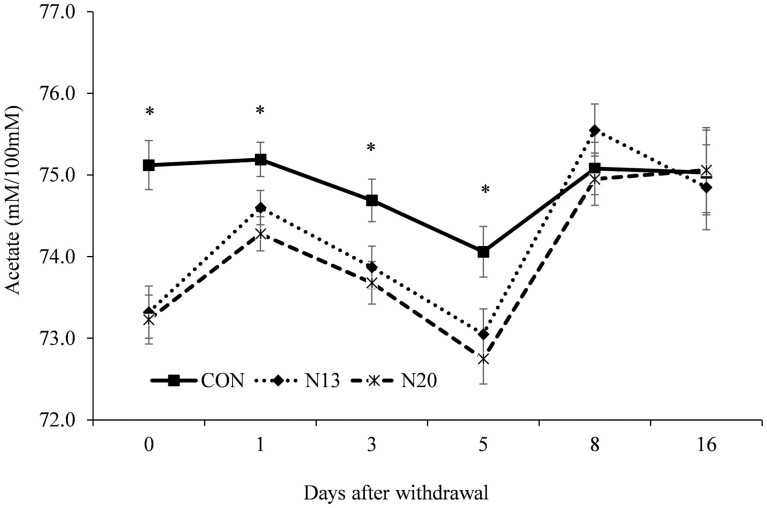
Acetate molar proportion during the narasin withdrawal period (16 d) of Nellore steers (*Bos indicus*) fed forage-based diet (*Cynodon dactylon* spp.) supplemented or not (**CON**; *n* = 10) with 13 mg of narasin/kg of DM (N13; *n* = 10), or 20 mg narasin/kg DM (**N20**; *n* = 10) for 140 d before narasin withdrawal. Day 0 represents the last day of narasin supplementation as described by Polizel et al. (2020).

**Figure 3.**
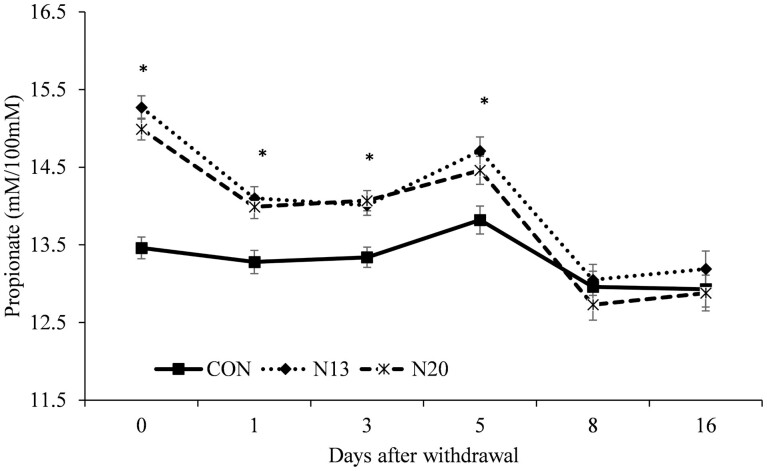
Propionate molar proportion during the narasin withdrawal period (16 d) of Nellore steers (*Bos indicus*) fed forage-based diet (*Cynodon dactylon* spp.) supplemented or not (**CON**; *n* = 10) with 13 mg of narasin/kg of DM (N13; *n* = 10), or 20 mg narasin/kg DM (**N20**; *n* = 10) for 140 d before narasin withdrawal. Day 0 represents the last day of narasin supplementation as described by Polizel et al. (2020).

**Figure 4.**
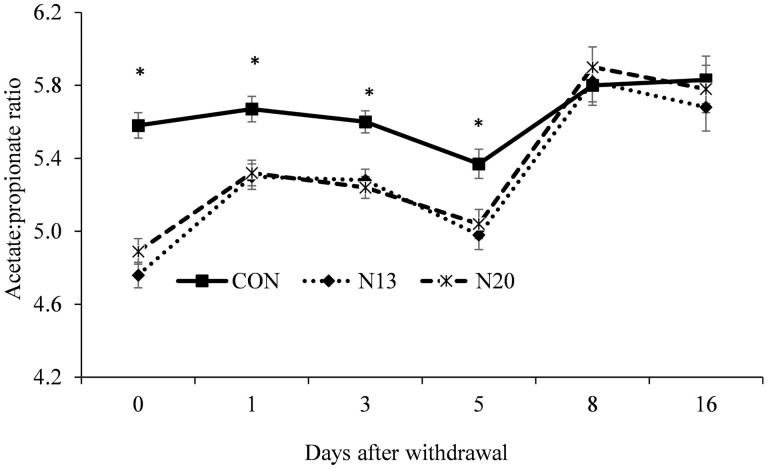
Acetate:propionate ratio during the narasin withdrawal period (16 d) of Nellore steers (*Bos indicus*) fed forage-based diet (*Cynodon dactylon* spp.) supplemented or not (**CON**; *n* = 10) with 13 mg of narasin/kg of DM (N13; *n* = 10), or 20 mg narasin/kg DM (**N20**; *n* = 10) for 140 d before narasin withdrawal. Day 0 represents the last day of narasin supplementation as described by Polizel et al. (2020).

**Figure 5.**
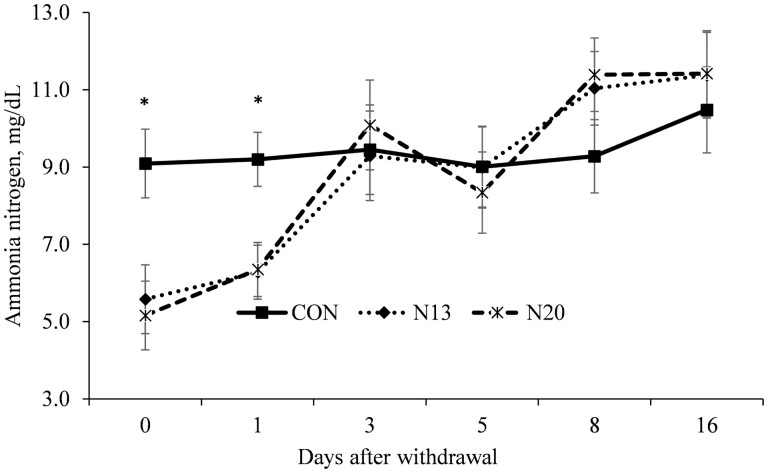
Ruminal ammonia nitrogen concentration during the narasin withdrawal period (16 d) of Nellore steers (*Bos indicus*) fed forage-based diet (*Cynodon dactylon* spp.) supplemented or not (**CON**; *n* = 10) with 13 mg of narasin/kg of DM (N13; *n* = 10), or 20 mg narasin/kg DM (**N20**; *n* = 10) for 140 d before narasin withdrawal. Day 0 represents the last day of narasin supplementation as described by Polizel et al. (2020).

A treatment × day interactions was detected (*P* ≤ 0.03) for the molar proportion of acetate (Figure 2), propionate (Figure 3), ac:prop ratio (Figure 4), and rumen ammonia nitrogen (Figure 5). Animals fed narasin maintained the molar proportion of acetate lower than the control animals until 5 d after withdrawal (*P* < 0.01; Figure 2), with no effect being observed on days 8 and 16 of the study (*P* > 0.07). The inclusion of narasin maintained the molar proportion of propionate greater than the control until 5 d after removal of narasin from the diets (*P* ≤ 0.04; Figure 3), whereas there was no effect on days 8 and 16 (*P* > 0.23). The use of narasin linearly decreased the ac:prop ratio up to 5 d after removal of the additive from the diets (*P* < 0.01; Figure 4), with no effect on days 8 and 16 (*P* > 0.45). Narasin linearly decreased ammonia N up to 1 d after withdrawal (*P* < 0.01; Figure 5), and there was no effect on days 3, 5, 8, and 16 (*P* > 0.13).

## DISCUSSION

The prolonged effect of feed additives after diet withdrawal was recently questioned due to the impact that these results may have on the nutritional management of animals, directly affecting the animal’s productivity (Limede et al., 2021). This residual effect facilitates the understanding of the positive effects of these molecules even when consumption occurs erratically, a reality observed in animals raised on pasture receiving feed additives through mineral and protein supplements (Cappellozza et al., 2018). In addition, the long-term effects of these additives can directly influence studies carried out in reversal design (Latin square, crossover, change-over), demonstrating a mandatory need for a minimum withdrawal period and minimizing possible carryover effects. Recent studies have evaluated the residual effect of monensin for cattle (Bell et al., 2017) and narasin for lambs (Pasqualino et al., 2020), but there is no study in the literature evaluating the withdrawal effects of narasin on beef cattle.

Although narasin has similar results to monensin in handling ruminal fermentation parameters and nutrient digestibility (Polizel et al., 2021), data in the literature show that narasin does not impact DMI in high-concentrate (Polizel et al., 2021) or forage-based diets (Limede et al., 2021; Polizel et al., 2020). These results suggest that narasin might be a favorable nutritional tool in multiple supplements, as DMI has a high-positive correlation with animal performance. Previous studies have shown that monensin did not change the DMI after withdrawal in cattle (Bell et al., 2017; Rogers et al., 1997). Pasqualino et al. (2020) also reported no impact on the DMI of lambs after narasin withdrawal. Additionally, Soares et al. (2021) described that supplementation with narasin daily, every 2 or 3 d, even with the adjustment of the supply dose, the total DMI was not affected. Polizel et al. (2020) demonstrated no effect of the total feed intake during the supplementation period, regardless of the dose (13 or 20 ppm), and the data from the present study complement these data by demonstrating that the abrupt interruption of narasin supply also does not change the intake of steers fed forage-based diets.

The variation in DMI observed over the days (Figure 1) of the experiment may be related to factors intrinsic to the animal’s diet and metabolism. High forage diets are characterized by low-energy value (Mertens, 1987), and voluntary DMI may be limited by rumen distension resulting from restriction of digest flow through the gastrointestinal tract (Allen, 1996). In addition, body conditions, meal size, and dietary particle sizes can alter the time spent in rumination, affecting the next meal (Mertens, 1996). The next day’s intake is variable and impacted by the day the animal does not ingest the adequate amount of energy to supply energy maintenance, and this variation can be extended over the days (Pasqualino et al., 2020).

The prolonged effect on the molar proportion of propionate observed in the present study had already been mentioned in the literature for cattle receiving narasin and fed with high forage content (Limede et al., 2021; Polizel et al., 2020; Soares et al., 2021). The main effect of including ionophores in the diet is altering fermentation dynamics in the rumen toward increased propionate, which is the primary glucose precursor for ruminants (NASEM, 2016), and is a critical hydrogen drain, reducing the availability of this compound for methane synthesis (Johnson and Johnson, 1995). Additionally, propionate concentration increases when an ionophore is included in diets as succinate-to-propionate reducing bacteria are resistant to the effects of the ionophore (McGuffey et al., 2001). Experiments evaluating the residual effect of ionophores on ruminal propionate demonstrate a prolonged action capacity of these dietary additives after withdrawal (Bell et al., 2017; Pasqualino et al., 2020). Bell et al. (2017) reported that propionate concentration was higher in animals previously fed with monensin until day 7 after removal. The use of narasin for lambs fed diets containing high forage content resulted in a persistent effect on propionate up to 4 d after removing the molecule from the diet (Pasqualino et al., 2020). Based on the literature and the data obtained in the present study, it is possible to infer that the molecule has a persistent residual effect on rumen fermentation parameters. These results are important, as they can help in decision-making regarding the nutritional management of animals used in experimental studies and production systems, where the intake of supplements might be erratic (commonly observed in animals receiving low-intake supplement) or due to the frequency of supplementation (Cappellozza et al., 2018).

The inclusion of ionophores into beef cattle diets alters SCFA dynamics by changing ruminal bacterial communities, resulting in more efficient fermentation pathways (Prange et al., 1978), increasing energy recovery from hexoses (Raun, 1990), and metabolizable energy availability (Tedeschi et al., 2003). The inclusion of narasin into the diet reduced the molar concentration of acetate in concentrate- (Polizel et al., 2021) or forage-based diets (Limede et al., 2021; Polizel et al., 2020). Bell et al. (2017) observed that acetate concentration remained lower after monensin withdrawal than in control diets until day 7. The result obtained in the present study shows that in animals subjected to long periods of exposure to narasin (140 d), even after removal of the ionophore from the diet, there is still a residual effect, maintaining the lowest molar proportions of acetate for up to 5 d after removal of the narasin.

The persistent increase in propionate and decrease in acetate in the rumen environment can explain the decrease in the ac:prop ratio. This result is consistent with previous studies that reported a reduction in the ac:prop ratio when narasin was offered to beef cattle (Limede et al., 2021; Polizel et al., 2020, 2021). Accordingly, the inclusion of monensin or narasin into beef cattle or lamb diets, also resulted in a lower ac:prop ratio from 4 to 7 d after withdrawal (Bell et al., 2017; Pasqualino et al. 2020).

Narasin was expected to change the molar proportion of butyrate, as reported by previous studies (Polizel et al., 2020, 2021). The decrease in butyrate can be partially justified by the alteration in the microbial population, inhibiting the growth of two different strains of *Butyrivibrio* (Dennis et al., 1981). Polizel et al. (2021) related that monensin and narasin resulted in a decrease in ruminal butyrate in lambs fed with a high-concentrate diet when compared to the control treatment; however, monensin showed greater reducing capacity when compared to narasin. Bell et al. (2017) observed the residual effect of monensin in decreasing the concentration of butyrate. Pasqualino et al. (2020) reported that the removal of narasin did not result in a residual effect on butyrate, a result similar to that observed in the present study, suggesting once again that monensin is possibly more potent in reducing butyrate compared to narasin.

The total SCFA concentration is affected by several factors, such as digest passage rate, saliva dilution, and water intake, water movement through the ruminal epithelium, SCFA production, and absorption by the epithelium (Leng and Brett, 1966). The total SCFA represents 75% to 85% of the fermentable energy, with the remainder being lost in the form of heat and methane (Sutton, 1979). There is no consensus in the literature on the effects of narasin on ruminal SCFA concentration. In diets with high forage content, the inclusion of ionophore increased the total SCFA concentration (Oliveira et al., 2022; Polizel et al., 2020), while in diets with high concentrates, the total SCFA concentration decreases (Polizel et al., 2021). In agreement with the results reported by Pasqualino et al. (2020), the present study demonstrated that there is no residual effect of narasin on the SCFA concentration in the rumen fluid after the removal of the ionophore.

The degradation of protein in the rumen is considered an inefficient process, as the amount of ammonia generated is greater than the capacity of the utilization by the microorganisms (Yang and Russell, 1993). In this scenario, ionophores play an important role by decreasing the amount of ammonia in the rumen (Goodrich et al., 1984), especially by decreasing the deamination process, and increasing the influx of amino acids into the gut (Chen and Russel, 1991). However, the presence of ammonia in the rumen is of great importance for some microorganisms, therefore, the reduction in deamination promoted by ionophores should not result in a deficit of ruminal nitrogen. Fibrolytic bacteria use ammonia as the preferred source of nitrogen (Nagaraja, 2016), and ruminal ammonia concentration below 5 mg/dL often limits microbial growth and affects fermentation (Slyter et al., 1979). In the present study, despite the residual effect of narasin reducing ammonia in the rumen fluid up to 1 d after removal, none of the current evaluations the value was <5 mg/dL. In a previous study, Pasqualino et al. (2020) reported a reduction in ammoniacal N up to 3 d after the removal of narasin and, as observed in the present study, none of the evaluations the ammonia concentration was lower than the minimum necessary for bacterial growth.

## CONCLUSION

The use of narasin for a prolonged period (140 d; Polizel et al., 2020) results in a residual effect in the rumen fermentation process after the removal of the additive from the diets. The residual effects observed in the ruminal parameters are commonly altered by the inclusion of narasin (propionate, ac:prop ratio, total SCFA, and ammoniacal N), and this effect can extend up to 5 d after narasin withdrawal. The results presented here have a great practical appeal, demonstrating that the residual effect of the molecule can help to circumvent supplementation management failures, as well as the natural fluctuation in the consumption of supplements by animals, without losing the beneficial effect of the molecule on the ruminal fermentation parameters. In addition, such information is also relevant to academic research. The data obtained in the present study demonstrate that it is necessary for at least 5 d of washout between the experimental periods so that there is no effect on the parameters of rumen fermentation when narasin is used.
